# Protective Efficacy of a Chimeric Insect-Specific Flavivirus Vaccine against West Nile Virus

**DOI:** 10.3390/vaccines8020258

**Published:** 2020-05-29

**Authors:** Laura J. Vet, Yin Xiang Setoh, Alberto A. Amarilla, Gervais Habarugira, Willy W. Suen, Natalee D. Newton, Jessica J. Harrison, Jody Hobson-Peters, Roy A. Hall, Helle Bielefeldt-Ohmann

**Affiliations:** 1 School of Chemistry and Molecular Biosciences, University of Queensland, St Lucia, Queensland 4072, Australia; l.vet@uq.edu.au (L.J.V.); y.setoh@uq.edu.au (Y.X.S.); a.amarillaortiz@uq.edu.au (A.A.A.); willy.suen@csiro.au (W.W.S.); natalee.newton@uq.net.au (N.D.N.); j.harrison1@uq.edu.au (J.J.H.); j.peters2@uq.edu.au (J.H.-P.); 2 School of Veterinary Science, University of Queensland Gatton Campus, Queensland 4343, Australia; g.habarugira@uq.net.au; 3 Australian Infectious Diseases Research Centre, University of Queensland, St Lucia, Queensland 4072, Australia

**Keywords:** West Nile virus, insect-specific flavivirus, chimeric flavivirus, vaccine

## Abstract

Virulent strains of West Nile virus (WNV) are highly neuro-invasive and human infection is potentially lethal. However, no vaccine is currently available for human use. Here, we report the immunogenicity and protective efficacy of a vaccine derived from a chimeric virus, which was constructed using the structural proteins (prM and E) of the Kunjin strain of WNV (WNV_KUN_) and the genome backbone of the insect-specific flavivirus Binjari virus (BinJV). This chimeric virus (BinJ/WNV_KUN_-prME) exhibits an insect-specific phenotype and does not replicate in vertebrate cells. Importantly, it authentically presents the prM-E proteins of WNV_KUN_, which is antigenically very similar to other WNV strains and lineages. Therefore BinJ/WNV_KUN_-prME represents an excellent candidate to assess as a vaccine against virulent WNV strains, including the highly pathogenic WNV_NY99_. When CD1 mice were immunized with purified BinJ/WNV_KUN_-prME, they developed robust neutralizing antibody responses after a single unadjuvanted dose of 1 to 5 μg. We further demonstrated complete protection against viremia and mortality after lethal challenge with WNV_NY99_, with no clinical or subclinical pathology observed in vaccinated animals. These data suggest that BinJ/WNV_KUN_-prME represents a safe and effective WNV vaccine candidate that warrants further investigation for use in humans or in veterinary applications.

## 1. Introduction

West Nile virus (WNV) is an arthropod borne flavivirus that mainly circulates between bird populations and its natural mosquito host, *Culex* spp [1,2,3]. Occasionally, a virus can spill over and cause infections in humans, an inadvertent host. Although mostly asymptomatic, WNV infections can cause a range of symptoms in humans, from mild febrile illness to more severe diseases such as paralysis and meningitis [4]. In 1999, WNV caused a major outbreak of fever and encephalitis in New York City. This particularly virulent strain of WNV, named WNV_NY99_, caused an unusually high rate of neurological symptoms with 63% of the patients developing encephalitis and a 12% mortality rate [5,6]. Apart from the occasional human outbreaks, horses are known to incur serious WNV infections, representing 96.9% of all mammalian cases caused by WNV [7,8,9]. Like humans, horses are dead-end hosts, as the viremia is not sufficient to sustain transmission to mosquitoes [10]. Several vaccines have been developed and licensed for equine use, but so far there are still none licensed for use in humans [11]. It is crucial for a vaccine to be both safe and highly effective. One of the major concerns about sub-unit and inactivated vaccines is low immunogenicity, which usually has to be complemented with a strong adjuvant to induce the required antibody response and usually requires frequent re-vaccinations. On multiple occasions, this has been linked to unwanted allergic reactions [12]. Live-attenuated vaccines are highly effective and, in most cases, eliminate the need for an adjuvant. However, these bring higher risk of the virus reverting to virulence, thus making them inappropriate for use in humans who are immunocompromised [13,14,15].

Previously, we reported the generation of BinJ/WNV_KUN_-prME, a chimeric flavivirus that encodes the structural prME genes of WNV_KUN_ on the genetic backbone of the insect-specific flavivirus (ISF) Binjari virus (BinJV) nonstructural protein genes [16]. During vertebrate infection, the flavivirus envelope (E) proteins engage with cellular receptors leading to virus internalization and replication. To prevent this, virus neutralization by antibodies directed to the EDIII receptor-binding domain of the virus is one of the requirements for the host to be protected [17,18,19]. We previously demonstrated that BinJ/WNV_KUN_-prME authentically presents all E protein epitopes, including those in EDIII, when compared to the wildtype WNV_KUN_. BinJ/WNV_KUN_-prME chimera can be produced to high titers in insect cells but exhibits an insect-specific phenotype and is unable to replicate in vertebrate cells. This provides a critical element of safety in the context of its assessment as a vaccine. Unlike previously reported chimeric flavivirus vaccines based on YFV or DENV backbones, the inability of the BinJ/WNV-prME chimeric virus to replicate in vaccinated individuals, eliminates any risk of reversion to virulence and thus would be more suitable for use in immunocompromised people and pregnant women.

Here, we report the assessment of immunogenicity and efficacy of BinJ/WNV_KUN_-prME as a novel WNV vaccine candidate and demonstrate protection of mice against lethal challenge with the virulent WNV_NY99_ strain. In addition, we show that further inactivation treatment of this vaccine does not adversely influence epitope presentation or protection in vivo.

## 2. Materials and Methods

### 2.1. Animal Ethics Statement

All animal work was carried out in accordance with the “Australian Code for the Care and Use of Animals for Scientific Purposes” as defined by the National Health and Medical Research Council of Australia. All experiments had received approval by the University of Queensland Animal Ethics Committee (permits SCMB/008/18 and SCMB/361/17). Three- to six-week-old CD1 mice were purchased from the Animal Resources Centre, Murdoch, Western Australia.

### 2.2. Cell Culture

C6/36 (*Aedes albopictus*) mosquito cells were grown in RPMI 1640 medium supplemented with 5% fetal bovine serum (FBS) at 28 °C. Vero African green monkey (*Cercopithecus aethiops*) kidney cells were maintained in Dulbecco’s modified Eagle medium (DMEM) with 5% FBS and were incubated at 37 °C with 5% CO_2_. All media was supplemented with penicillin (50 U/mL), streptomycin (50 μg/mL) and 2 mM L-glutamine (PSG).

### 2.3. Generation of BinJ/WNV_KUN_-prME

RNA extraction, Circular Polymerase Extension Reaction and transfection to obtain the BinJ/WNV_KUN_-prME chimera was previously described [16].

### 2.4. Viruses

BinJV was isolated in our laboratory from Ae. normanensis mosquitoes as previously reported in Hobsen-Peters et al. [16]. The NY99-4132 strain of WNV was obtained from the Division of Vector-Borne Infectious Diseases, Centers for Diseases Control, Fort Collins, CO, USA [20]. The KUNV-NSW2011 strain of WNV was isolated in Australia from horse brains at the Elizabeth Macarthur Agriculture Institute, Menangle, New South Wales, Australia [21]. 

#### Monoclonal Antibodies

All monoclonal antibodies and their general characteristics that were used in this study have been reported previously [16]. WNV-specific monoclonal antibodies used were 3.91D, 3.67G, 2B2, 10A1 (anti-WNV E) and P10F8 (anti-WNV-prM). Antibodies BJ-6E6, BJ-1E1, 4G4 were cross-reactive between BinJV and vertebrate-infecting flaviviruses (VIFs). VIF-specific antibodies used in this study were 4G2 and M2-1E7, which were not able to detect BinJV.

### 2.5. Enzyme-linked Immunosorbent Assay (ELISA)

Plates (detailed in 2.5.1 and 2.5.2) were blocked in 150 μL ELISA blocking buffer [0.05M tris-HCl (pH 8.0), 1 mM EDTA, 0.15 M NaCl, 0.05% (v/v) Tween 20 and 0.2% (w/v) casein] for 1 h at room temperature. Heat-inactivated mouse serum (56 °C/30 min; 1/50 dilution in blocking buffer) was added to first column, titrated out across the plate in doubling dilutions (for total IgG fixed-cell ELISA) or primary monoclonal antibody hybridoma supernatant (coating ELISA; Section 2.5.2) and incubated at 37 °C with 5% CO_2_ for 1 h, with a total volume of 50 μL per well [16]. After 3 washes with PBS-T, 50 μL/well horseradish peroxidase-labelled goat anti-mouse Ig was added and incubated at 37 °C with CO_2_ for 1 h. Five PBS-T washes were performed before adding 100 μL substrate buffer [1 mM 2,2-azino- bis(3-ethylbenzthiazoline-6-sulfonic acid) and 3 mM H_2_O_2_ in substrate buffer [0.1 M citric acid with 0.2 M Na_2_HPO_4_ (pH 4.2)]]. Substrate was incubated at room temperature for 1 h in the dark. ODs were measured at 405 nm in spectrophotometer.

#### 2.5.1. Fixed-Cell ELISA Plate Preparation

C6/36 cells were seeded at 10^4^ cells per well in a 96-well plate (Corning Incorporated Costar). The following day, cells were infected with WNV_KUN_ at a multiplicity of infection (MOI) of 0.1 and fixed 5 days post-infection in fixative buffer (20% acetone with 0.02% bovine serum albumin (BSA) in PBS).

#### 2.5.2. Antigen-Coated ELISA on Purified Chimeric Virus

Live or inactivated purified BinJ/WNV_KUN_-prME virions were diluted in PBS to a final concentration of 80 ng per well. One-hundred microliters per well was added to high-binding 96-well plates (Greiner) and left to bind overnight at 4 °C. The plates were washed 3 times in PBS-T prior to blocking.

### 2.6. Vaccine Production and Purification

C6/36 cells were pre-seeded in T175 culture flasks and infected with BinJ/WNV_KUN_-prME a MOI 0.1, 3 days post-seeding. Virus inoculum was prepared in RPMI with 2% FBS, and PSG, and 4 mL were added per flask. Cells were incubated for 1 h at room temperature while rocking. Virus inoculum was then discarded and 20 mL of fresh RPMI media with 2% FBS and PSG were added. Cells were incubated at 28 °C. Virus supernatant was collected at 3 dpi and clarified at 10,000× *g* for 30 min in a tabletop centrifuge to remove any cell debris. Fresh media was added back onto infected cells and harvesting was repeated every 2 days, for a maximum of 5 harvests. Clarified supernatant was stored at 4 °C until virus purification.

#### 2.6.1. Vaccine Purification

Polyethylene glycol 6000 (40% PEG6000 in NTE) was added to the virus supernatant in a 1:4 ratio. The solution was stirred slowly overnight at 4 °C. Virus-PEG solution was then centrifuged at 12,000× *g* for 2 h at 4 °C to pellet the virus. The pellet was resuspended in 5 mL cold NTE buffer [120 mM NaCl, 10 mM Tris, 1 mM EDTA (pH 8.0)]. A 40% sucrose cushion was layered under the virus suspension and centrifuged at 28,000 ×*g* for 2 h at 4 °C. The virus pellet was resuspended in 250 μL NTE buffer and buffer exchanged into PBS using 100 kDa centricon tubes. Purified virus was stored at 4 °C.

#### 2.6.2. Quantification of Vaccine

Purified BinJ/WNV_KUN_-prME was run on an unreduced SDS-PAGE along with a pre-made BSA standard curve (0.1, 1, 2, 2.5, 3, 3.5 and 10 mg/mL). Band intensity was determined using Fiji-ImageJ software and yield of E monomer was resolved by comparison to BSA standard [16]. 

### 2.7. UV Inactivation of BinJ/WNV_KUN_-prME Vaccine

To determine optimal UV-C exposure time, purified BinJ/WNV_KUN_-prME dose was prepared in PBS in a 6-well plate and placed on ice in the biosafety cabinet. The lid was removed from the plate and the vaccine was exposed to UV-C for different lengths of time. The optimal exposure time was determined by performing a TCID_50_ assay to ensure that no infectious virus remained [20]. Data was extrapolated based on the TCID_50_ results to determine the time required for complete inactivation. For vaccine preparation, purified BinJ/WNV_KUN_-prME vaccine was exposed to UV-C for 90 min and then stored at 4 °C until time of vaccination. A TCID_50_ assay was performed before vaccination to ensure inactivation.

### 2.8. Micro-Neutralization Assay of Mouse Serum Samples

Micro-neutralization assays were performed using Vero cells. Mouse sera were heated to 56 °C for 30 min to inactivate complement. Serum samples were added in 96-well plates, starting at a 1 in 20 dilution, then titrated out in doubling dilutions. Hundred infectious units of wildtype virus (WNV_NY99_ or WNV_KUN_) was added per well and the virus-serum mixture incubated for 1 h at 37 °C + 5% CO_2_ before adding 10^4^ Vero cells per well. The culture plates were incubated for 5 days and then fixed in 20% acetone with 0.02% bovine serum albumin (BSA) in PBS. Neutralizing antibody titers were determined in a fixed-cell ELISA (Section 2.5 and Section 2.5.1) using 4G4 monoclonal antibody.

### 2.9. Vaccination of CD1 Mice with BinJ/WNV_KUN_-prME

Vaccine doses were prepared in PBS no more than 1 h before vaccination and kept on ice. All vaccines were administered subcutaneously at the base of the tail using a 27-gauge insulin needle, with a maximum volume of 50 μL. Booster doses were given 21 days apart. Animals were monitored after vaccination for potential adverse effects.

#### 2.9.1. Assessment of Immunogenicity of BinJ/WNV_KUN_-prME with and without Advax^TM^

Six-week old male and female CD1 mice were immunized with either 1 or 5 μg BinJ/WNV_KUN_-prME with half of the mice receiving a booster dose. Vaccination groups included: 1 × 1 μg, 2 × 1 μg, 1 × 5 μg, 2 × 5 μg, each group either with or without Advax^TM^ (Vaxine Pty Ltd., Adelaide, Australia) and a non-vaccinated PBS control (*n* = 5 per group, either 2 males and 3 females or 3 males and 2 females). Adjuvanted doses of BinJ/WNV_KUN_-prME with Advax^TM^ were prepared within 2 h of vaccination by combining the vaccine with Advax^TM^ (1mg/mouse) in PBS. Mice were tail bled at 4, 8, 16 and 26 weeks post-last vaccination by making a minor incision in the tail. Blood was collected in BD Microtainer^®^ (Becton, Dickinson & Co., Franklin Lakes, NJ, USA) gel tubes and was allowed to settle for 30 min before serum was separated by centrifugation for 10 min at 11,000 x g. Sera were stored at −20 °C. The majority of male mice were culled prior to week 26 due aggressive behavior. Therefore, only complete data sets are presented in this study for four- and eight-weeks post-vaccination. Antibody titers were determined in fixed cell ELISA (with WNV_KUN_) and micro-neutralization assays.

#### 2.9.2. Vaccination with Live and UV Inactivated BinJ/WNV_KUN_-prME

Three-week old male and female CD1 mice were immunized with 1 or 5 μg purified BinJ/WNV_KUN_-prME in PBS, either live virus or UV-inactivated. Mice vaccinated with 1 μg received a booster dose after 21 days. Vaccination groups included: 2 × 1 μg dose, 1 × 5 μg dose, 2 × 1 μg dose UV, 1 × 5 μg dose UV and a PBS control group (*n* = 10 per group, 5 males and 5 females). Tail bleeds were collected 2 days before booster and virus challenge (Section 2.9.1).

### 2.10. Virus Challenge with WNV_NY99_

WNV_NY99_ was propagated in C6/36 cells by infecting a confluent monolayer with MOI 0.01. Virus was incubated for 1 h at 28 °C before removing inoculum and adding fresh RPMI with 2% FBS and PSG. After 5 days, the supernatant was replaced with fresh media to minimize the occurrence of defective interfering particles in the virus stock [22]. New supernatant was harvested the following day and clarified by centrifugation to remove cell debris. Virus was stored in RPMI with 10% FBS at −80 °C. Virus was titrated using the TCID_50_ method on Vero cells and fixed 5 dpi [23].

#### 2.10.1. WNV_NY99_ Challenge in CD1 Mice

Mice were infected intraperitoneally with 10^3^ infectious units of WNV_NY99_ 21 days post-vaccination. Mice were monitored twice daily for any clinical signs and weighed once daily. To determine viremia levels, tail bleeds were taken daily, with each individual mouse bled no more than once every three days. Animals were humanely euthanized when weight loss was ≥20% of initial body weight and/or if animal had severe clinical signs, including but not limited to a depressed appearance, abdominal swelling or neurological signs. After virus challenge, animals were culled when reaching a humane endpoint as stipulated by the AEC permit or at the end of the trial on day 21 post-infection. A final cardiac bleed was performed on animals under deep anesthesia (50 mg/kg of ketamine and 10 mg/kg of xylazine). Following cervical dislocation, the head and spines were collected from all animals and fixed in 10% neutral-buffered formaldehyde for 24–36 h, after which the tissues were transferred to 70% ethanol until further processing. Additional tissues (intestines, liver, spleen, kidneys, lung, heart) were also collected and processed from animals experiencing clinical signs of disease. Heads and spines were decalcified in 8% formic acid in water (*v/v*) for 4–5 days and then trimmed and subjected to routine processing for paraffin-embedding. Five µm sections were stained with hematoxylin and eosin (H and E) and examined on a Nikon Eclipse 51 E microscope. Digital microphotographs were taken using a Nikon DS-Fi1 camera with a DS-U2 unit and NIS elements F 4.60 software. Images are reproduced without manipulations other than cropping and adjustment of light intensity. Immunohistochemistry for detection of flavivirus NS1 protein in tissues was performed on 4–5 µm FFPE sections as previously described in detail [24].

#### 2.10.2. Viremia Titrations in Immune-Plaque Assay (IPA)

Blood obtained via tail bleeds were centrifuged to separate serum and frozen at −80 °C. C6/36 cells were pre-seeded at a concentration of 10^5^ cells per well in a 96-well plate. Sera were titrated out in RPMI with 2% FBS and PSG in tenfold serial dilutions and transferred onto pre-seeded cells (25 μL per well). Cells were incubated to infect for 2 h at 28 °C, 5% CO_2_, before removing the inoculum and adding 200 μL of overlay media (2X M199 medium supplemented with 5% FBS, PSG and 2% carboxymethyl cellulose. Media was removed from the cells 48 h after infection and cells were fixed in 100 μL 80% ice-cold acetone in PBS at −20 °C for 30 min, then air-dried. For probing, plates were blocked with 100 μL KPL milk diluent/blocking solution in PBS-T at 37 °C for 1 h. Fifty microliters of purified hE16 [25,26] antibody (1μg/mL) was added and incubated for 1 h at 37 °C to label for WNV E protein. Plates were washed 3 times in PBS-T before adding 50 μL of secondary antibody (IRDye 800CW goat anti-human IgG, LI-COR at 12.5 ng/well) and incubated at 37 °C for 1 h. After five PBS-T washes, plates were air-dried and scanned on Odyssey Clx reader with the following specifications: channel = 800, intensity = auto, resolution = 42 μm and focal length = 3mm. Resulting viral titers are shown as foci forming units per mL (FFU/mL) [27].

### 2.11. Statistical Analysis

Statistical analysis was performed using Prism 8 for MacOS (version 8.4.2 (464) 2020). Multiple *t*-tests were used for analysis of IgG and neutralizing antibody titers. For weight loss comparison, repeated-measures ANOVA analysis of variance was used. Survival curve was analyzed using a Mantel–Cox (log-rank) test. 

## 3. Results

### 3.1. Immunogenicity of BinJ/WNV_KUN_-prME is Adequate without an Adjuvant

**Figure 1:**
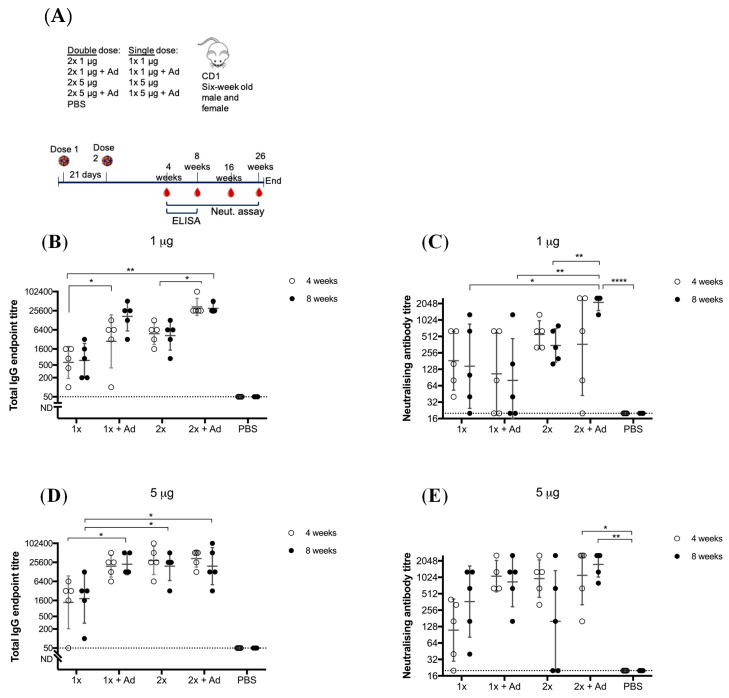
Vaccination of adult male and female CD1 mice with Binjari virus (BinJ)/West Nile virus (WNV)_KUN_-prME with and without Advax^TM^ (Ad). Adult CD1 mice (*n* = 5) were immunized subcutaneously on the rump with purified BinJ/WNV_KUN_-prME in PBS. (**A**) Vaccination timeline. Geometric mean of total IgG antibody response and neutralizing antibody titers after vaccination with 1 μg (**B**,**C**) or 5 μg (**D**,**E**) purified BinJ/WNV_KUN_-prME. Antibody levels were measured by fixed-cell Enzyme-linked Immunosorbent Assay (ELISA) on WNV_KUN_-infected C6/36 cells using the 4G4 monoclonal antibody (**B**,**D**) and by micro-neutralization assay with wildtype virus strain WNV_KUN_ on Vero cells (**C**,**E**). Error bars represent SD of the geometric mean. Limit of detection is represented by dotted line at 1/20 dilution. Multiple *t*-test statistical analysis was performed to determine statistical significance (* *p* < 0.0032, ** *p* < 0.0021, **** *p* < 0.0001).

To determine whether this vaccine requires the need for an adjuvant to confer adequate immunity, we compared BinJ/WNV_KUN_-prME given with or without Advax^TM^. Advax^TM^ is a polysaccharide adjuvant derived from delta inulin and has been proven to enhance antibody responses as well as lower the required vaccination dose, leading to antigen sparing [28,29]. CD1 mice received one or two doses of either 1 or 5 μg of purified BinJ/WNV_KUN_-prME, with or without Advax^TM^, given by the subcutaneous route (Figure 1).

Animals given a single dose of 1 μg of BinJ/WNV_KUN_-prME vaccination without adjuvant, developed a robust immune response of both total IgG (geometric mean (GM) titer of 900, range 100–1600 at 4 weeks and GM titer of 1200, range 200–3200 at eight weeks (Figure 1B) and neutralizing antibody levels (mean GM titer of 312, range 40–640 at four weeks and GM titer of 416, range 20–1280 at eight weeks (Figure 1C)). When a booster dose was given, total IgG levels increased, although not significantly. The mean neutralizing antibody titer increase was also not significant, however individual titers doubled for three out of five animals. When doses were given with Advax^TM^, significant increase was seen for IgG levels, both for a single and a double dose. Neutralizing antibodies only significantly increased in the 2 × 1 μg + Ad group. When the dose of vaccination was increased to 5 μg, we observed a robust response in IgG levels (mean GM titer was 2890, with a range of 50–6400 for four weeks post-vaccination and GM titer of 4180, with a range of 100–12,800 at eight weeks (Figure 1D)) and neutralizing antibody response (mean GM titer of 188, range 20–400 at four weeks and GM titer of 680, range 40–1280 at eight weeks (Figure 1E)). For both 1 μg and 5 μg doses there was a significant increase in total IgG response when formulated with adjuvant, although no significant increase was observed for the neutralizing antibody titer in the respective groups. For 5 μg doses, both a booster vaccination and/or adding adjuvant, significantly increased IgG levels. However, the 1 × 5 μg + Ad, 2 × 5 μg and 2 × 5 μg + Ad groups were not statistically different when compared to each other, indicating some form of dose saturation had been reached. No significant difference was observed for total IgG levels at four- and eight-weeks post-immunization, indicating that IgG levels were maintained. Furthermore, at 16- and 26-weeks post-immunization the antibody titers remained steady, indicating a long-lasting response for at least six months (Figure S1). All vaccinated mice developed neutralizing antibodies of >20, independent of vaccine group, suggesting that although an adjuvant increases neutralizing antibody titers, it is not essential for inducing a robust immune response. No difference was observed between male or female mice.

### 3.2. BinJ/WNV_KUN_-prME Stability

**Figure 2:**
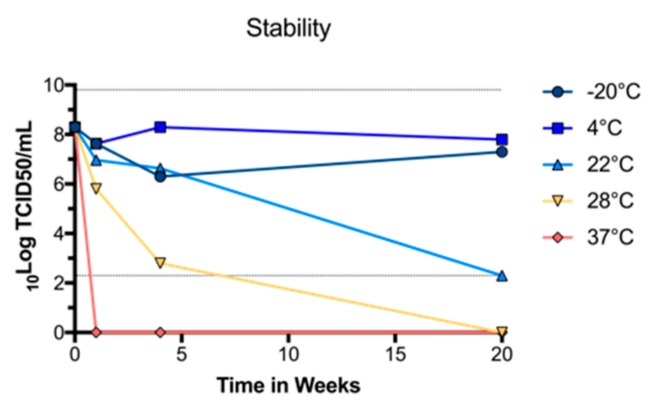
Stability of purified BinJ/WNV_KUN_-prME in PBS when stored at different temperatures. Infectious virus titers were determined by TCID_50_ assay at one, four and twenty weeks. Dotted lines at 2.3 and 9.8 Log_10_TCID_50_/mL represent upper and lower limits of detection, respectively.

To determine whether purified BinJ/WNV_KUN_-prME was stable at different temperatures, we diluted the vaccine Preparation in PBS and incubated at −20 °C, 4 °C, room temperature (~22 °C), 28 °C or 37 °C, for either 1, 4 or 20 weeks. The vaccine was then titrated in a TCID_50_ assay, using C6/36 cells to determine the titer of infectious virus. We observed no drop in infectious virus titer up until four weeks when incubated at room temperature or lower (Figure 2). While the infectious virus titers dropped rapidly after incubation at 37 °C, or even at 28 °C, the virus was stable at 4 °C for up to 20 weeks, indicating that this vaccine can be effectively stored in a refrigerator.

### 3.3. UV-Treated BinJ/WNV_KUN_-prME Is Antigenically Authentic

**Figure 3:**
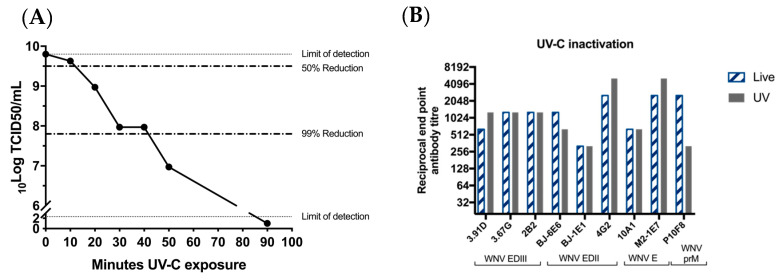
UV-treatment of BinJ/WNV_KUN_-prME does not affect authentic epitope presentation. (**A**) Infectious BinJ/WNV_KUN_-prME titer in TCID_50_/mL after 10, 20, 30, 40, 50 or 90 min of UV-C exposure. UV-treated vaccine was inoculated onto C6/36 and infectious virus titer was determined in fixed cell ELISA probed with 4G4. Dotted lines represent 50% and 99% reduction of infectious virus. Limits of detection are 2.3 log_10_TCID_50_/mL (lower limit) and 9.8 log_10_TCID_50_/mL (upper limit). (**B**) Epitope authenticity was determined by binding of monoclonal antibodies to prM and E domains and end-point dilutions were determined by ELISA on live versus UV-C inactivated chimeric virus antigen.

Despite the inability of the chimeric virus vaccine to replicate in vertebrate cells, our recent data indicate that it is capable of entering vertebrate cells and delivering the RNA genome into the cytoplasm [30]. It is also likely that initial (but undetectable) rounds of viral translation and dsRNA synthesis occur before replication is aborted by vertebrate innate immune mechanisms [30]. Based on the potent immune response generated by a single low dose of the vaccine in the absence of adjuvant (reported here and previously [16]), we suspect that a self-adjuvating effect may be due to immune recognition of low levels of viral dsRNA by RIG-1 and production of inflammatory cytokines (TNF alpha; IL6, etc.) that enhance the humoral response. To ablate any low-level viral translation and transcription that might occur after entry of the chimeric virus to a vertebrate cell, BinJ/WNV_KUN_-prME was exposed to UV-C. This causes pyrimidine dimerization and uridine hydrates which renders the RNA as an unsuitable template for translation and/or transcription [16]. The purified vaccine was exposed to UV-C for 10, 20, 30, 40 or 50 min, to determine the exposure time required to render the virus replication-deficient in mosquito cells. Virus titrations performed after the exposure times showed a ≥99.85% reduction in infectious virus titer after 50 min of UV-C exposure (Figure 3A). For the vaccine preparation to be administered to the mice, we used a 90-min exposure time to ensure complete inactivation of the vaccine virus. We then showed that authentic epitope presentation was unaffected by this treatment, using a range of monoclonal antibodies, including M21E7, which recognizes a highly conformational epitope (Figure 3B).

### 3.4. BinJ/WNV_KUN_-prME Vaccination Protects CD1 Mice against Viremia and Clinical Signs of Disease after Lethal Challenge with WNV

**Figure 4:**
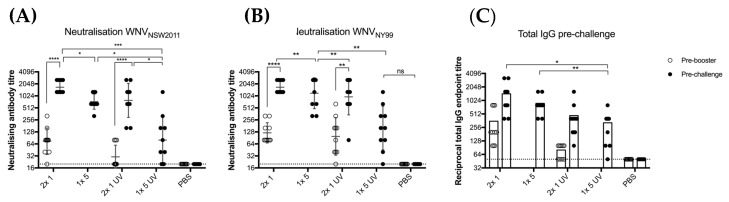
Vaccination of CD1 mice with untreated versus UV-treated BinJ/WNV_KUN_-prME vaccine. Male and female adult CD1 mice (*n* = 5 of each) were vaccinated with either 1 or 5 μg of purified BinJ/WNV_KUN_-prME (untreated or UV-treated), or PBS (mock) vaccination. Vaccinations were administered subcutaneously on the rump, with booster doses given 21 days apart. Tail bleeds were collected before administering the booster dose (pre-booster) or 20 days post-booster (pre-challenge). Neutralizing antibody titers were determined on wildtype viruses for both KUN (**A**) and NY99 strains (**B**) of WNV on Vero cells, fixed at 5 dpi. Limit of detection is shown as dotted line at 1 in 20 dilution. (**C**) Total IgG endpoint titers determined in fixed cell ELISA with C6/36 cells infected with wildtype WNV_KUN_, using 4G4 antibody. Limit of detection is shown as dotted line at 1 in 50 dilution. Error bars represent SD of the geometric mean. Statistical analysis was performed using multiple *t*-test (* *p* < 0.0032, ** *p* < 0.0021, *** *p* < 0.0002, **** *p* < 0.0001).

Based on the antibody levels observed in the previous experiment, we aimed to assess the protective immunity induced by untreated and UV-treated BinJ/WNV_KUN_-prME. Total IgG levels and neutralizing antibody levels were determined after vaccination with UV-treated or untreated BinJ/WNV_KUN_-prME (Figure 4). Mice received either two doses of 1 μg (2 × 1 μg) or a single dose of 5 μg (1 × 5 μg) of purified BinJ/WNV_KUN_-prME, either live of UV-C treated. Although all mice developed a neutralizing antibody response after a double dose of 1 μg or a single dose of 5 μg, antibody levels observed in mice vaccinated with UV-treated BinJ/WNV_KUN_-prME were significantly lower than with the untreated chimera. This suggests that the presence of viable viral RNA may play a role in the high antibody responses observed after even a single vaccination with a BinJV-based chimeric vaccine. Neutralizing antibody titers were determined against both WNV_KUN_, which was used to generate this chimera, and the highly pathogenic WNV_NY99_ strain that causes severe disease in humans, horses and birds. No difference was observed in neutralization levels against the two strains of WNV, demonstrating that the BinJ/WNV_KUN_-prME chimera induced a strong cross-reactive antibody response to a heterologous WNV strain, similar to our results with other WNV_KUN_-based vaccines [20,31].

**Figure 5:**
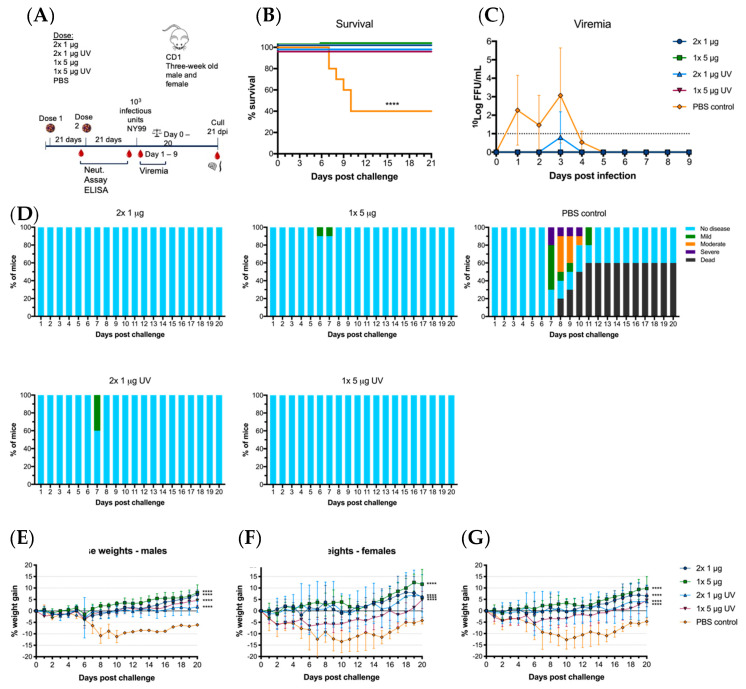
Virus challenge with WNV_NY99_ after vaccination with live of UV-C inactivated BinJ/WNV_KUN_-prME vaccine. Male and female adult CD1 mice were challenged intraperitoneally with 10^3^ infectious units of WNV_NY99_ at 21 days-post vaccination (*n* = 10; five males and five females). (**A**) Timeline of vaccination and bleeding. (**B**) Survival shown for all mice for 21 days-post virus challenge. (**C**) Mean viremias were determined by IPA on C6/36 cells, fixed at 2 dpi and probed with hE16 anti-WNV E [26]. Limit of detection was 1 log_10_FFU/mL for individual serum samples. (**D**) Clinical signs of disease were collected for 21 days following infection by monitoring animals twice daily. Colors represent severity of clinical signs as a proportion of total animals in the group. Mouse weights were recorded daily and are represented as mean percentage of weight change from day of infection per group (**G**), or males (**E**) or females (**F**). Error bars represent SD of the mean.

Immunized mice were then challenged with 10^3^ infectious units of WNV_NY99_ at 21 days post-vaccination at the age of 12 weeks. Within 12 days post-infection, 60% of control (PBS-vaccinated) mice succumbed to the virus, with the remaining 40% showing milder clinical signs and significant weight loss (Figure 5D–G). Of the vaccinated animals, 100% survived until 21 dpi with only one animal in the 1 × 5 μg UV-C group showing low levels of viremia on day three post-challenge (Figure 5B,C). This animal only showed mild clinical signs of disease and weight loss remained within the acceptable range, as did a few other animals in this group (Figure 5D–G). Mice in the remaining vaccine groups (2 × 1 μg, 1 × 5 μg and 2 × 1 μg UV-C) gained weight during the 21 days-post challenge and only 5 out of 30 animals showed mild ruffled fur (Figure 5D). WNV_NY99_ viremia was detected in all control animals with virus detected from day one to four. We observed no direct correlation between high virus titer and mortality in individual animals. A difference was observed for weight loss between males and females, with males losing less weight than females (Figure 5E,F). These results did not translate to a difference between males and females in clinical signs or mortality. All vaccinated groups had significantly less weight loss than the unvaccinated control mice (*p* < 0.0001).

### 3.5. BinJ/WNV_KUN_-prME Vaccination Protects against Sub-Clinical Pathology

It was observed that infected male mice in the PBS control group were gaining weight after challenge while the females were rapidly losing weight. However, there was no difference observed between male and female mortality. Several of the animals, which were terminated due to severe clinical signs or weight loss, had severely distended abdomen and intestines, notably jejunum and ileum, with 3 out of 10 animals in the control group having erosive/necrotizing enteritis and severe ulcerative gastritis as seen microscopically. Viral antigen was detected in neurons of myenteric ganglia of these mice, suggesting that the dilation may be due to ileus. None of these observations were made in the vaccinated mice.

Microscopic examination of the brain and spinal cords revealed mild to moderate meningo-encephalitis and/or myelitis in 8 out of 10 PBS vaccinated mice, with one mouse showing loss of a substantial numbers of neurons in the hippocampus. Viral antigen was detected in the brain of five of these animals. Vaccinated mice, whether receiving untreated or UV-treated chimeric vaccine, were devoid of histopathological changes in the brain and spinal cord at the time of termination at 21 dpi. As the four PBS-vaccinated mice that survived the WNV challenge showed substantial neurological damage, but no clinical neurological signs, it may be surmised that the vaccine protected against not just clinical disease but also subclinical pathology and potential long-term sequelae.

## 4. Discussion

Here, we demonstrate that a BinJ/WNV_KUN_-prME chimeric vaccine induces high and long-lasting levels of virus neutralizing antibody responses that protect mice against lethal challenge with a virulent WNV strain. Despite being non-infectious for vertebrates, the vaccine showed good efficacy after a single dose in the absence of an adjuvant. This supports our previous studies that showed similar protective efficacy of a BinJ/ZIKV-prME vaccine in a mouse model of ZIKV disease [16].

We also previously reported that BinJ/WNV_KUN_-prME and BinJ/ZIKV-prME particles exhibit authentic epitope presentation compared to the wild type pathogen [16]. Thus, it is likely that the structural and antigenic integrity of the BinJ/WNV_KUN_-prME vaccine is at least partially responsible for the potent protective immunity we have demonstrated here. However, as reported previously, we also hypothesize that these BinJV-chimeric vaccines may have an inherent self-adjuvating capability, possibly caused by initial synthesis of viral replicative dsRNA intermediates upon entry of the vertebrate cell before replication is aborted by innate responses and other mechanisms [16,30]. To investigate this further, we showed that after inactivation of the purified BinJ/WNV_KUN_-prME vaccine with UV-C, despite significantly lower neutralizing antibody titers compared to untreated vaccine (*p* < 0.0021), immunized mice were still protected from lethal challenge and significant clinical signs of disease. This is consistent with our demonstration that UV inactivation did not significantly alter the epitope presentation. Hence, even though this vaccine is non-infectious to vertebrates, if issues arise in regard to licensing of a GMO-vaccine, there is a viable option for inactivating the chimeric vaccine with little compromise to efficacy.

Despite the robust protective responses induced in the absence of an adjuvant, we did observe a significantly enhanced response in some vaccine groups when the vaccine was formulated with the polysaccharide Advax^TM^ adjuvant. Advax^TM^ is a GMP-grade delta-inulin polysaccharide-based adjuvant that has been shown to induce a balanced Th1/Th2 immune response to human vaccines in a number of clinical trials with minimal adverse reactions [28,29]. Indeed, we also show that, when formulated with an inactivated Japanese encephalitis virus (JEV) vaccine preparation, Advax^TM^ induces a robust neutralizing antibody response against JEV in horses and is well tolerated in foals and pregnant mares [32]. These results suggest that, should further dose sparing be required for a BinJ/WNV_KUN_-prME vaccine, Advax^TM^ provides a suitable adjuvant formulation for further studies.

Our findings also warrant further comparisons of safety and efficacy of the BinJ/WNV_KUN_-prME vaccine with other WNV vaccines that have been approved for veterinary use or have been assessed as candidates for a human WNV vaccine in terms of safety and efficacy. Two inactivated vaccines are currently approved for use in horses; Innovator (Pfizer) and Vetera^®^ WNV (Boehringer Ingelheim). Both require two doses four to six weeks apart with recommended booster doses every 6–12 months. A recombinant canary pox-vectored vaccine (Recomibitek^®^, Merial) is also given as two doses four to six weeks apart. To date, no WNV vaccines have been approved for use in humans. However, three candidate vaccines have entered clinical trials. The live-attenuated ChimeriVax-WNV vaccine, which uses the YFV-17D backbone to present the structural WNV prM-E genes, and a similar chimeric vaccine based on the DENV-4 genome backbone (WN-DEN4). Both vaccines were well tolerated and induced significant neutralization titers after a single dose [11]. A DNA-based, non-infectious vaccine expressing the WNV prM-E proteins has also been assessed in phase I trials. This vaccine was given as three doses and was well tolerated [33,34]. It is clear that our results with the BinJV-based chimeric vaccine platform eliminates a number of safety concerns associated with replicating vaccines, thereby providing a distinct advantage over live-attenuated chimeric vaccines. Furthermore, the induction of immunity after a single, unadjuvanted dose provides a favorable initial comparison with existing inactivated and recombinant vaccines.

The ability of this chimeric virus to replicate to high titers in insect cells will be a distinct advantage for large scale manufacturing and efficient vaccine production. Furthermore, the inability of the BinJ/WNV_KUN_-prME vaccine to replicate in vertebrate cells provides a significant safety aspect to allow propagation of the vaccine in low biocontainment facilities. However, further research is required to generate cell banks of mosquito cell lines for GMP approval. Further studies are also required to investigate whether prior vaccination with a BinJV-based chimera has any effect on efficacy of other BinJV-based vaccines that are subsequently given.

Because BinJ/WNV_KUN_-prME replicates exceptionally well in mosquito cell culture, we also acknowledge the remote possibility of wild mosquito populations acquiring and subsequently transmitting the vaccine virus. However, preliminary studies have shown that *Culex annulirostris*, a major mosquito vector of arboviruses in Australasia, cannot transmit BinJ/WNV_KUN_-prME, even when fed very high doses of the virus in a blood meal. This suggests that there is negligible risk in this regard. We are currently undertaking further studies with other important mosquito species for publication elsewhere.

## 5. Conclusions

In conclusion, we report a BinJV-based chimeric vaccine that presents the authentic epitopes of WNV and induces a protective antibody response against challenge with a lethal strain of the virus after a single unadjuvanted immunization. The inability of the vaccine to replicate in vertebrates provides a strong safety profile that would allow for its use in individuals normally contraindicated for live vaccines and its manufacture in minimal bio-containment facilities. Furthermore, we show that, even after UV-inactivation, the vaccine continued to provide protective efficacy, thus providing an option for a further level of safety and regulatory compliance if needed.

## Supplementary Materials

The following are available online at https://www.mdpi.com/2076-393X/8/2/258/s1, Figure S1: Vaccination of adult male and female CD1 mice with BinJ/WNV_KUN_-prME with and without Advax^TM^ (Ad). Adult CD1 mice (*n* = 5) were immunized subcutaneously on the rump with purified BinJ/WNV_KUN_-prME in PBS. Geometric mean of neutralizing antibody titers 16- and 26-weeks after vaccination with 1 μg (A) or 5 μg (B) purified BinJ/WNV_KUN_-prME. Antibody levels were measured by micro-neutralization assay with wildtype virus strain WNV_KUN_ on Vero cells. Error bars represent SD of the geometric mean. Statistical analysis was performed using multiple *t*-test (* *p* < 0.0032, ** *p* < 0.0021, *** *p* < 0.0002, **** *p* < 0.0001).
