# Supplementary material for: Protective Efficacy of a Chimeric Insect-Specific Flavivirus Vaccine against West Nile Virus
Source: Vaccines (Basel). 2020 May 29;8(2):258. doi: 10.3390/vaccines8020258 (PMC7349994; doi:10.3390/vaccines8020258)
Supplement: Supplementary file 1 [file vaccines-08-00258-s001.pdf]

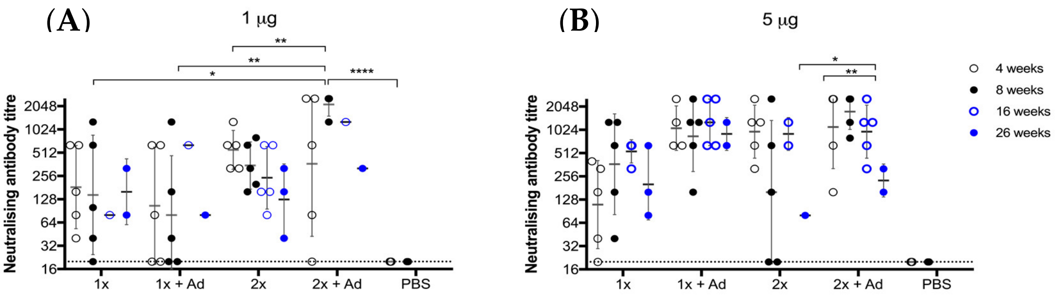

Figure S1. Vaccination of adult male and female CD1 mice with BinJ/WNV$_{KUN}$-prME with and without Advax^{TM} (Ad). Adult CD1 mice ($n = 5$) were immunized subcutaneously on the rump with purified BinJ/WNV$_{KUN}$-prME in PBS. Geometric mean of neutralizing antibody titers 16- and 26-weeks after vaccination with 1 μg (A) or 5 μg (B) purified BinJ/WNV$_{KUN}$-prME. Antibody levels were measured by micro-neutralization assay with wildtype virus strain WNV$_{KUN}$ on Vero cells. Error bars represent SD of the geometric mean. Statistical analysis was performed using multiple *t*-test (* $p < 0.0032$, ** $p < 0.0021$, *** $p < 0.0002$, **** $p < 0.0001$).
